# Choroidal vascularity changes in idiopathic central serous chorioretinopathy after half-fluence photodynamic therapy

**DOI:** 10.1371/journal.pone.0202930

**Published:** 2018-08-27

**Authors:** Dae Joong Ma, Un Chul Park, Ei Tae Kim, Hyeong Gon Yu

**Affiliations:** 1 Department of Ophthalmology, College of Medicine, Seoul National University, Seoul, Republic of Korea; 2 Retinal Degeneration Research Laboratory, Seoul National University Hospital Biomedical Research Institute, Seoul, Republic of Korea; National Yang-Ming University Hospital, TAIWAN

## Abstract

**Purpose:**

This study evaluated changes in choroidal vascularity after half-fluence photodynamic therapy (HF-PDT) in patients with central serous chorioretinopathy (CSC) using swept-source optical coherence tomography (SS-OCT) en face imaging.

**Methods:**

This retrospective comparative case series included 50 eyes of 25 patients with unilateral CSC who underwent HF-PDT and 50 age-and sex-matched normal healthy control eyes. En face SS-OCT images of the choriocapillaris, Sattler’s layer, and Haller’s layer were converted into binary images. The vascular proportions were defined as the percentage of the area of vascular lumen against the area of the 3.0-mm-diameter circular area. The main outcome measures were the vascular proportions before HF-PDT and at 6 weeks, 6 months, and 12 months after HF-PDT.

**Results:**

At baseline, the vascular proportions in the CSC eyes were significantly greater than those in the control eyes in all layers (choriocapillaris: 51.8% ± 15.5% vs. 41.3 ± 18.7%, P = 0.018; Sattler’s: 58.6% ± 13.4% vs. 49.7% ± 15.7%, *P* = 0.017; Haller’s: 65.3% ± 15.3% vs. 53.0% ± 13.4%, *P* = 0.001). In the CSC eyes, the vascular proportion in the choriocapillaris significantly decreased at 6 weeks (36.6% ± 16.9%, *P* < 0.001), 6 months (34.0% ± 12.3%, *P* < 0.001), and 12 months (34.8% ± 17.6%, *P* < 0.001) after HF-PDT compared with baseline. The vascular proportions in Sattler’s and Haller’s layers did not show a significant decrease at 6 weeks (Sattler’s: 49.7% ± 17.3%, *P* = 0.052 and Haller’s: 58.3% ± 12.9%, *P* = 0.558) but decreased significantly at 6 months (Sattler’s: 48.9% ± 12.4%, *P* < 0.001 and Haller’s: 57.7% ± 15.7%, *P* = 0.027) and 12 months after HF-PDT from the baseline values (Sattler’s: 45.8% ± 10.4%, *P* < 0.001 and Haller’s: 56.8% ± 15.7%, *P* < 0.001).

**Conclusion:**

After HF-PDT, the choriocapillaris showed the earliest decrease in vascular proportion of en face images, Sattler’s and Haller’s layers showed later decreases. The temporal differences in the response of each layer may reflect the pathophysiology of CSC and the therapeutic mechanism of HF-PDT.

## Introduction

Central serous chorioretinopathy (CSC) is a relatively common disorder characterized by serous detachment of the neurosensory retina at the posterior pole with points of leakage at the level of the retinal pigment epithelium (RPE) [1]. Abnormalities in choroidal vasculature, including congestion of the choroidal vessels and choroidal hyperpermeability, are associated with the pathogenesis of CSC [2]. Although no standard therapy exists, many studies suggest that photodynamic therapy (PDT) is an effective treatment for CSC [3].

Swept-source optical coherence tomography (SS-OCT) uses a long-wavelength light source of 1050 nm and a tunable laser, offering improved capacity to penetrate more deeply into tissues [4]. SS-OCT improves signal strength in the choroid and the choroid-sclera interface, which provides better resolution of the choroidal layers and coronal views of different depth levels (en face images). A recent study assessed choroidal vascular changes in eyes with CSC using en face SS-OCT imaging and reported that the choroidal vascular areas at the microvasculature of the inner choroid and at the large choroidal vessels were larger in eyes with CSC than those in age-matched normal eyes [5]. However, changes in the choroidal vasculature of CSC after PDT at different depth levels are unknown.

The purpose of this study was to clarify the changes in the choroidal vasculature in eyes with CSC and to evaluate their changes after half-fluence PDT (HF-PDT) using en face SS-OCT imaging. To accomplish this goal, we quantitatively assessed the choroidal vascular proportions before and after HF-PDT in the choriocapillaris, Sattler’s layer, and Haller’s layer using binarized en face SS-OCT images.

## Materials and methods

### Patient examination and treatment protocols

All patients who underwent HF-PDT to treat idiopathic CSC and were followed up with SS-OCT from December 2013 to November 2015 at Seoul National University Hospital (SNUH) were retrospectively reviewed. The Institutional Review Board (IRB) of SNUH approved this retrospective study, which was conducted in accordance with the Declaration of Helsinki. Patient records/information were anonymized and de-identified prior to analysis. The IRB waived the requirement for informed consent.

Patients were diagnosed with CSC when they exhibited subretinal fluid involving the macula with or without serous RPE detachment on OCT imaging and associated idiopathic leaks on fluorescein angiography (FA) and abnormal, dilated choroidal vasculature with hyperpermeability on indocyanine green angiography (ICGA) [3]. Patients with any evidence of choroidal neovascularization (CNV) or branching networks of choroidal vessels with aneurysm-like dilatations consistent with polypoidal choroidal vasculopathy were excluded from the diagnosis of CSC. Patients older than 18 years and with a diagnosis of active CSC for at least 3 months at the time of HF-PDT were included. Patients with systemic steroid use, pregnancy, Cushing's syndrome, end-stage renal disease, collagen vascular disease, *Helicobacter pylori* infection, obstructive sleep apnea, or organ transplantation were excluded. Patients with other macular abnormalities, insufficient image quality, or bilateral CSC were also excluded from the analysis. All patients with CSC underwent a comprehensive ophthalmic examination, which included best-corrected visual acuity, slit-lamp biomicroscopy, dilated funduscopy, spectral-domain OCT (Carl Zeiss Meditec Inc., Dublin, CA), SS-OCT (DRI OCT-1; Topcon Corp., Tokyo, Japan), FA (Kowa VX-10i; Kowa Company, Ltd., Tokyo, Japan), and ICGA (Heidelberg Retina Angiography; Heidelberg Engineering, Heidelberg, Germany). SS-OCT imaging was performed before (baseline) and at 6 weeks, 6 months, and 12 months after HF-PDT. When treating CSC eyes, HF-PDT (25 J/cm^2^, 689-nm laser, 83-second treatment time) with a full verteporfin dose was performed.

The PDT spot size was determined primarily based on the ICGA findings, as described in our previous work [6], comprising the smallest circle covering the choroidal hyperpermeability area on ICGA that was attributed to the accumulation of submacular fluid. In addition, any definite fluorescein leakage on FA presumed to be associated with submacular fluid was included in the PDT irradiation area.

To increase the statistical power of the present analysis [7], we recruited 50 healthy controls by retrospective medical chart review based on propensity score matching using the covariates of age and sex. Patient records/information were anonymized and de-identified prior to analysis. IRB waived the requirement for informed consent.

### Evaluation of vascular proportion, subfoveal choroidal thickness and choroidal vascularity index

**Fig 1:**
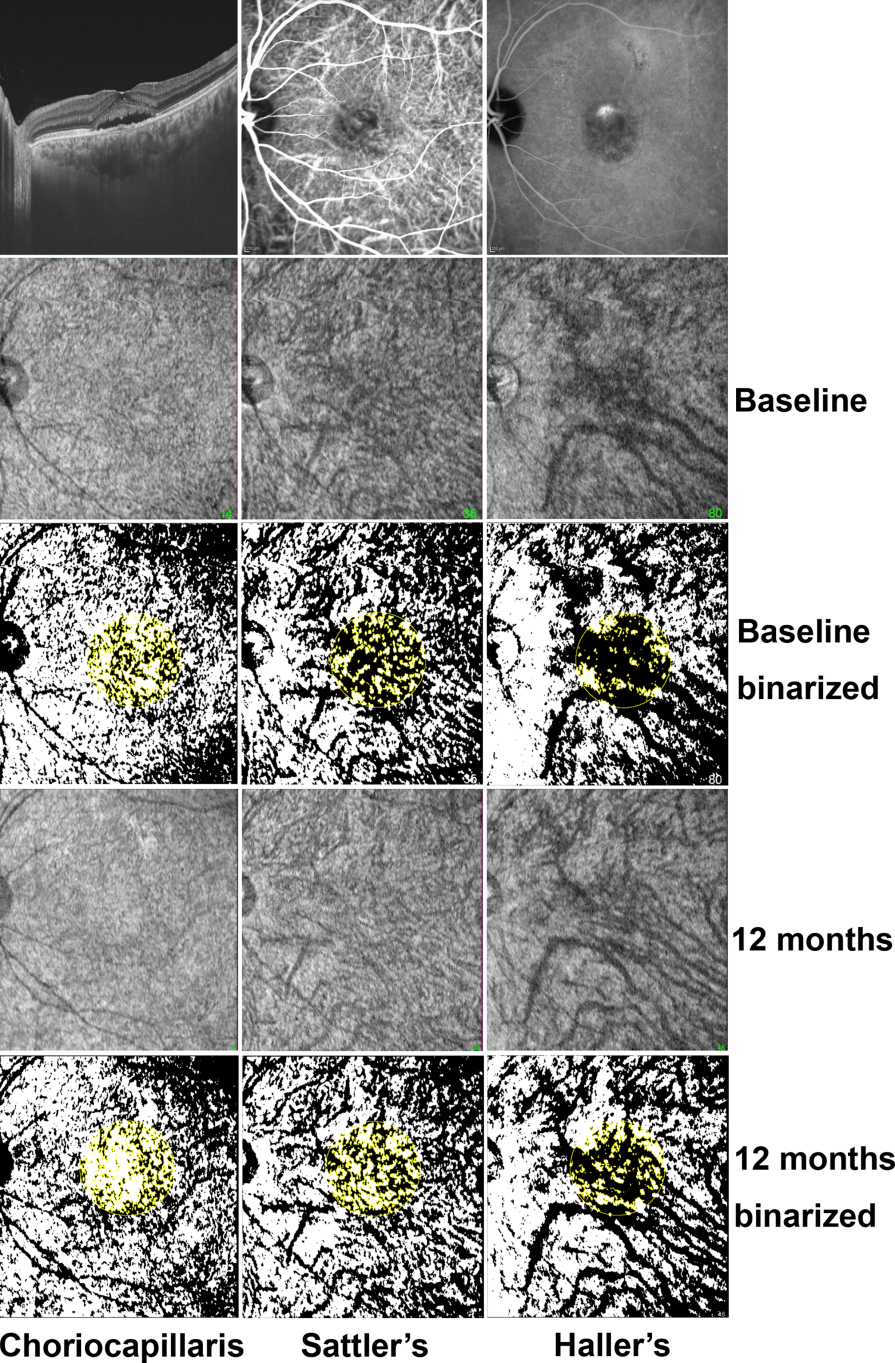
Representative images of the right eye of a 51-year-old woman with active central serous chorioretinopathy. An optical coherence tomography image shows serous retinal detachment. The hyperpermeable region is demonstrated in indocyanine green angiography. En face images at the level of the choriocapillaris, Sattler’s layer, and Haller’s layer before and 12 months after half-fluence photodynamic therapy (HF-PDT) were adapted and converted to binary images to distinguish the vascular lumen (black area) and choroidal stroma (white area). The choroidal vascular proportion was measured as the percentage of the area of the vascular lumen against the 3.0-mm-diameter area centered at the fovea. The binarized en face images showed decreased choroidal vascular proportions at all levels 12 months after HF-PDT.

The scan protocol was a 3-dimensional volumetric macular scan 12 × 9 mm area containing 512 × 256 axial scans. To measure vascular proportion, representative en face images of each vascular layer, which showed the most prominent vascular markings under the guidance of a B-scan, were obtained. En face images of the choroid were constructed at a depth of every 2.6 μm from the Bruch membrane. Then, en face images were flattened at the level of the Bruch membrane, and a moving average filter was applied using software developed by the Topcon Corporation (IMAGEnet ® 6, Topcon Corporation, Tokyo, Japan). In cases where segmentation error occurred in the delineation of the Bruch membrane, manual correction was used before generating en face images. The choriocapillaris was defined as a thin layer in the inner choroid with a relatively homogeneous layer with medium reflectivity without hyporeflective vascular markings on the en face images. Sattler’s layer was defined as a thick layer of round or oval-shaped hyperreflective profiles with hyporeflective cores in the mid-choroid that presented with medium-sized hyporeflective vascular markings on the en face images. Haller’s layer was defined as a thick layer of oval-shaped hyperreflective profiles with hyporeflective cores in the outer choroid that presented with large-sized hyporeflective vascular markings on the en face images [8, 9]. Given the changes in choroidal thicknesses at the different time points, we obtained follow-up en face images with vascular patterns that were the most similar to the previously adopted images. The area of choroidal vasculature of the adopted images was measured using ImageJ software (National Institutes of Health, Bethesda, Maryland, USA). Images were converted to 8-bit format, followed by binarization using the auto threshold function with the default method to distinguish the vascular lumen (black area) and the choroidal stroma (white area). Next, the portion of vascular lumen in the 3.0-mm-diameter area centered at the fovea was calculated. The choroidal vascular proportion was defined as the percentage of the area of vascular lumen against the area of the 3.0-mm-diameter circle (Fig 1).

The subfoveal choroidal thickness (SCT) was manually measured with a built-in caliper tool, defined as the vertical perpendicular distance from the hyperreflective line of the Bruch membrane to the innermost hyperreflective line of the chorioscleral interface [9].

To measure choroidal area and choroidal vascularity index (CVI), the protocol described by Sonoda et al [10] was adopted with modifications. The raster scan passing through the fovea was adopted for the evaluation. The subfoveal choroidal area with a width of 1,500 mm (750 mm on either side of fovea) was selected and added to the region of interest (ROI) manager. Perpendicular lines to the Bruch membrane were drawn from the innermost point of 5 randomly selected large (≥100 μm) luminal areas within the ROI. A line parallel to the Bruch membrane at the distance of the average length of these 5 lines was defined as the border between the inner choroid and the outer choroid. The inner choroid included the choriocapillaris and Sattler’s layer, and the outer choroid included Haller’s layer. The image binarization was performed with ImageJ software using the Niblack autolocal threshold tool. Black pixels were considered as luminal area, and white pixels were considered as stromal area. CVI was defined as the percentage of the luminal area against the whole choroid area (luminal area + stromal area) and was calculated in the total choroid, the inner choroid, and the outer choroid, respectively. Two investigators (DJM and ETK) independently measured the SCT, the choroid area, and CVI, and the average value of these 2 measurements was used in subsequent analyses.

### Statistical analysis

All values are presented as the means ± standard deviations. The Student's and paired Student's t-tests were used to compare the clinical characteristics, the choroidal vascular proportion, SCT, and CVI among the CSC eyes, unaffected fellow eyes, and control eyes. The paired Student's t-test was used to compare the choroidal morphological parameters before and after HF-PDT. Bivariate relationships among the choroidal vascular proportion, SCT, and CVI were examined using the Pearson product-moment correlation coefficient test. All statistical analyses were performed using SPSS software for Windows, version 22.0 (IBM, Armonk, NY, USA). Propensity score matching was performed using an SPSS R Essentials plug-in (IBM) and R statistics version 2.15.0 (R Foundation for Statistical Computing, Vienna, Austria). A value of *P* < 0.05 was regarded as statistically significant.

## Results

In this study, 25 CSC eyes and 25 unaffected fellow eyes from 25 unilateral CSC patients and 50 control eyes from 50 healthy subjects were included in the analysis. The mean duration of active CSC before PDT treatment was 9.8 months. There were no significant differences in age, gender distribution, or refractive errors between the CSC patients and controls (Table 1).

**Table 1:** Baseline demographics, choroidal vascular proportions of en face images, subfoveal choroidal thicknesses, and choroidal vascularity index of B-scan images in studied eyes.

	Control Eyes (n = 50)	CSC Eyes (n = 25)	Fellow Eyes (n = 25)	*P*-value^a^	*P*-value^b^	*P*-value^c^
Age (y)	50.2 ± 7.5	50.9 ± 10.8	50.9 ± 10.8	0.753	0.753	N/A
Sex (M/F)	36/14	18/7	18/7	1.000	1.000	N/A
Spherical equivalent (diopters)	-0.5±1.5	-0.4±0.9	-0.3±1.1	0.855	0.668	0.410
Choroidal vascular proportion (%)						
Choriocapillaris	41.3 ± 18.7	51.8 ± 15.5	39.8 ± 18.3	0.018	0.734	0.003
Sattler’s layer	49.7 ± 15.7	58.6 ± 13.4	51.9 ± 12.0	0.017	0.546	0.064
Haller’s layer	53.0 ± 13.4	65.3 ± 15.3	60.5 ± 15.6	0.001	0.034	0.274
SCT (μm)	232.1 ± 77.7	379.4 ± 95.7	329.4 ± 103.7	< 0.001	< 0.001	0.007
Choroidal vascularity index (%)						
Total choroid	64.2 ± 3.4	65.6 ± 1.7	65.9 ± 2.7	0.018	0.036	0.524
Inner choroid	78.2 ± 10.1	73.3 ± 11.9	74.1 ± 13.2	0.068	0.138	0.858
Outer choroid	61.7 ± 3.8	64.9 ± 2.1	65.0 ± 3.2	< 0.001	< 0.001	0.710

CSC: central serous chorioretinopathy; SCT: subfoveal choroidal thickness.

^a^*P-*value comparing control eyes and eyes with CSC, Student's t-test.

^b^*P*-value comparing control eyes and unaffected fellow eyes, Student's t-test.

^c^*P-*value comparing eyes with CSC and unaffected fellow eyes, paired Student's t-test.

The choroidal vascular proportion of en face images at baseline was significantly higher in the CSC eyes in all choroidal vascular layers than in the control eyes (choriocapillaris: *P* = 0.018; Sattler’s: *P* = 0.017; Haller’s: *P* = 0.001; Table 1). In comparison, the vascular proportion in Haller’s layers was significantly higher in the unaffected fellow eyes than in the control eyes (*P* = 0.034); however, no differences were observed between the control eyes and unaffected fellow eyes in the choriocapillaris (*P* = 0.734) or Sattler’s layer (*P* = 0.546). In addition, the baseline vascular proportion was significantly greater in the CSC eyes than in the unaffected fellow eyes (*P* = 0.003) in the choriocapillaris but not in Sattler’s layer (*P* = 0.064) or Haller’s layer (*P* = 0.274).

The mean baseline SCT in the CSC eyes was significantly greater than those in both the control eyes (*P* < 0.001) and the unaffected fellow eyes (*P* = 0.007). The baseline SCT was greater in the unaffected fellow eyes than in the control eyes (*P* < 0.001).

The CSC eyes showed significantly higher CVI of B-scan images in the outer choroid (*P* < 0.001) and the total choroid (*P* = 0.018) than the control eyes but showed no difference in the inner choroid (*P* = 0.068, Table 1). However, no differences were observed between the CSC eyes and unaffected fellow eyes in all layers (total choroid: *P* = 0.524; inner choroid: *P* = 0.858; outer choroid: *P* = 0.710). Unaffected fellow eyes also showed significantly higher CVI in the outer choroid (*P* < 0.001) and the total choroid (*P* = 0.036) than the control eyes but showed no difference in the inner choroid (*P* = 0.138).

### Relationships among vascular proportion, subfoveal choroidal thickness, and choroidal vascularity index

**Fig 2:**
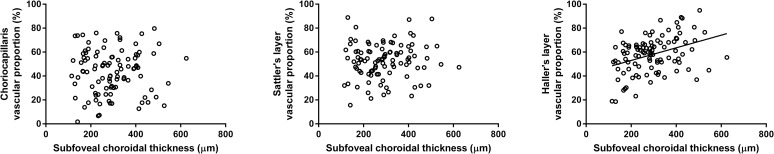
Relationship between subfoveal choroidal thickness and choroidal vascular proportion of en face images. (A) The choriocapillaris (r = 0.013, *P* = 0.901) and (B) Sattler’s layer (r = 0.079, *P* = 0.436) had no statistically significant correlation. (C) The vascular proportion of Haller’s layer (r = 0.383, *P* < 0.001) was directly proportional to the subfoveal choroidal thickness (Pearson product-moment correlation coefficient test).

The SCT was significantly correlated with the vascular proportions in Haller’s layer (r = 0.383, *P* < 0.001) but not with those in the choriocapillaris (r = 0.013, *P* = 0.901) or Sattler’s layer (r = 0.079, *P* = 0.436; Fig 2).

The CVI in the inner choroid was significantly correlated with the vascular proportions in the choriocapillaris (r = 0.274, *P* = 0.006) and Sattler’s layer (r = 0.281, *P* = 0.005). The CVI in the outer choroid was significantly correlated with the vascular proportions in Haller’s layer (r = 0.244, *P* = 0.014).

### Changes in vascular proportion, subfoveal choroidal thickness, and choroidal vascularity index after half-fluence photodynamic therapy

**Fig 3:**
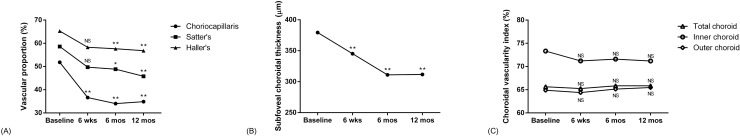
Changes in choroidal vascular proportions of en face images, subfoveal choroidal thickness, and choroidal vascularity index of B-scan images in CSC eyes after half-fluence photodynamic therapy (HF-PDT). (A) The vascular proportion in the choriocapillaris significantly decreased after HF-PDT (6 weeks: *P* < 0.001; 6 months: *P* < 0.001; 12 months: *P* < 0.001). The vascular proportions in Sattler’s and Haller’s layers did not show a significant decrease at 6 weeks (Sattler’s: *P* = 0.052 and Haller’s: *P* = 0.558) but decreased significantly at 6 months (Sattler’s: *P* < 0.001 and Haller’s: *P* = 0.027) and 12 months after HF-PDT (Sattler’s: *P* < 0.001 and Haller’s: *P* < 0.001). (B) The SCT decreased significantly after HF-PDT (6 weeks: *P* = 0.007; 6 months: *P* < 0.001; 12 months: *P* < 0.001). (C) The CVIs of the total choroid (6 weeks: *P* = 0.448; 6 months: *P* = 0.710; 12 months: *P* = 0.718), the inner choroid (6 weeks: *P* = 0.370; 6 months: *P* = 0.410; 12 months: *P* = 0.189), and the outer choroid (6 weeks: *P* = 0.434; 6 months: *P* = 0.653; 12 months: *P* = 0.354) showed no significant changes after HF-PDT. NS: not significant, *P < 0.05, ** *P* < 0.01; paired Student's t-test.

The mean PDT spot size was 3201.8 ± 927.5 μm. All PDT spot locations involved the 3.0-mm-diameter area centered at the fovea. In all patients, subretinal fluid accumulation disappeared completely at 6 weeks after HF-PDT and did not recur during the follow-up period. Within the 12 months after HF-PDT, there were no PDT-related complications, such as RPE atrophy, persistent choriocapillaris hypoperfusion, or secondary CNV. The vascular proportion in the choriocapillaris showed significant decreases at 6 weeks (36.6% ± 16.9%, *P* < 0.001), 6 months (34.0% ± 12.3%, *P* < 0.001), and 12 months (34.8% ± 17.6%, *P* < 0.001) after HF-PDT (Fig 3A). The vascular proportions in Sattler’s and Haller’s layers did not show significant decreases at 6 weeks (Sattler’s: 49.7% ± 17.3%, *P* = 0.052 and Haller’s: 58.3% ± 12.9%, *P* = 0.558) but decreased significantly at 6 months (Sattler’s: 48.9% ± 12.4%, *P* < 0.001 and Haller’s: 57.7% ± 15.7%, *P* = 0.027) and 12 months after HF-PDT (Sattler’s: 45.8% ± 10.4%, *P* < 0.001 and Haller’s: 56.8% ± 15.7%, *P* < 0.001). The SCT decreased significantly after HF-PDT (6 weeks: 345.2 ± 92.0 μm, *P* = 0.007; 6 months: 311.1 ± 77.6 μm, *P* < 0.001; 12 months: 311.6 ± 86.8 μm, *P* < 0.001; Fig 3B). However, the CVIs of the total choroid (6 weeks: 65.3% ± 2.8%, *P* = 0.448; 6 months: 65.8% ± 2.6%, *P* = 0.710; 12 months: 65.8% ± 2.7%, *P* = 0.718), the inner choroid (6 weeks: 71.2% ± 7.0%, *P* = 0.370; 6 months: 71.6% ± 8.5%, *P* = 0.410; 12 months: 71.2% ± 8.9%, *P* = 0.189), and the outer choroid (6 weeks: 64.4% ± 2.9%, *P* = 0.434; 6 months: 65.1% ± 2.6%, *P* = 0.653; 12 months: 65.5% ± 2.9%, *P* = 0.354) showed no significant changes after HF-PDT compared with baseline (Fig 3C).

The vascular proportions in the unaffected fellow eyes at 12 months after HF-PDT did not show significant changes (choriocapillaris: 38.5% ± 17.8%, *P* = 0.808; Sattler’s: 51.3% ± 16.9%, *P* = 0.770; Haller’s: 62.6% ± 15.9%, *P* = 0.830). At 12 months after HF-PDT, the SCT and the vascular proportion were not significantly different between the CSC eyes and the unaffected fellow eyes (SCT: *P* = 0.934; choriocapillaris: *P* = 0.476; Sattler’s: *P* = 0.134; Haller’s: *P* = 0.170).

## Discussion

In this study, we assessed the vascular proportion within a 3 mm-diameter circular area at the center of the fovea in 3 separate layers of the choroid using binarized SS-OCT en face imaging in unilateral CSC patients. All 3 layers (the choriocapillaris, Sattler’s layer, and Haller’s layer) showed increased vascular proportion in the CSC eyes compared with that in the control eyes but showed significant decreases after HF-PDT. The CSC eyes showed increased vascular proportion in the choriocapillaris compared with that in the unaffected fellow eyes, suggesting pathogenesis underlying the accumulation of subretinal fluid in CSC. The choriocapillaris showed the earliest and largest decrease in the vascular proportion, reflecting the short-term therapeutic response after HF-PDT, whereas Sattler’s and Haller’s layers showed later decreases that were suggestive of the long-term effects of HF-PDT.

The exact pathophysiology of CSC remains unknown. However, abnormalities in the choroidal vasculature, including congestion of choroidal vessels, choroidal hyperpermeability, and leakage into the choroidal stromal space, are presumed to be associated with the pathogenesis of CSC [1, 11–15]. Interestingly, we found that the vascular proportion of the choriocapillaris was significantly larger in the CSC eyes than the unaffected fellow eyes and the control eyes and was not significantly different between the unaffected fellow eyes and the control eyes. These findings are consistent with previous studies of CSC eyes with ICGA that showed capillary or venous congestion in the early or mid-phase [15] and suggest that capillary and venous congestion are the key mechanisms in CSC. Conversely, the increased vascular proportions of Haller’s layer may primarily contribute to the increased SCT in CSC patients, in both the affected eyes and unaffected fellow eyes. This finding suggests that this disorder may essentially be a bilateral or systemic disorder, which results in the dilatation of large choroidal vessels (Haller’s layer) and thickening of the choroid, not only in the active CSC eyes but also in the fellow eyes. Several studies evaluating choroidal vascular changes using various imaging modalities have reported findings consistent with those of the present study. Yang et al [16] evaluated the mean diameter of the largest hyporeflective lumen in Haller’s layer using OCT B-scan images. The mean diameters in the CSC eyes and unaffected fellow eyes were larger than that of the control eyes but were comparable between the CSC eyes and the unaffected fellow eyes in unilateral CSC patients. Chan et al [17] revealed a greater choriocapillaris width in CSC eyes than in unaffected fellow eyes using OCT angiography. However, OCT angiography has limitations in the evaluation of Sattler’s and Haller’ layers because of backscattering of light from the RPE–Bruch membrane complex, flow projection artifacts caused by the choriocapillaris, and interference fringe washout caused by high flow in the larger choroidal vessels [18, 19].

We also evaluated the CVI using B-scan images. The CVI gives combined information regarding the vasculature (luminal area) and the interstitial tissues (stromal area). Because the luminal and stromal areas were increased together in the CSC eyes, the CVI provides more intuitive information about CSC. Agrawal et al [20] reported that acute CSC eyes showed statistically significant increased CVI in comparison with control eyes and normal fellow eyes. Sonoda el al [10] evaluated CVIs of inner, outer, and whole choroid separately. The CSC eyes showed larger CVIs in the inner, outer, and total choroid than control eyes. These results were generally in accordance with our findings with the CVI (Table 1).

In the present study, the CVI of the inner choroid, which included the choriocapillaris and Sattler’s layer, correlated with the vascular proportion of the choriocapillaris and Sattler’s layer, and the CVI of the outer choroid, which included Haller’s layer, correlated with the vascular proportion of Haller’s layer. These findings suggest that the vascular proportions evaluated using en face images are in accordance with the CVIs evaluated using B-scan images, which have been demonstrated to have clinical value in previous studies [10, 20]. However, the CVI is not suitable for the objective of our study; the evaluation of the choroidal vasculature changes in 3 different choroid layers of CSC eyes after HF-PDT for the following reasons. First, it is challenging to clearly document the choroidal microvasculature with B-scan OCT imaging due to insufficient axial resolution for a small caliper and the complexity of the vascular network. Therefore, CVI cannot provide separate information for the choriocapillaris and Sattler’s layer [21, 22], which change in different ways after HF-PDT. Second, the CVI changes respond not only to the stromal area but also to the luminal area because the value is calculated as the luminal area divided by the sum of the luminal area and the stromal area, which decrease simultaneously after HF-PDT (S1 Appendix). This explains why the CVIs of the CSC eyes did not change after HF-PDT in the present study (Fig 3C).

To overcome this limitation of the CVI, we evaluated the choroidal vasculature changes using the vascular proportion obtained from the en face imaging, which has the following advantages. First, en face imaging provides more precise segmentation of the choriocapillaris, Sattler’s layer, and Haller’s layer due to the orientation of the images, which follows the anatomic distribution of the choroidal vessels. Second, the vascular proportion is calculated as the portion of vascular lumen in the 3.0-mm-diameter area centered at the fovea. This vascular proportion reflects solely choroidal vasculature changes and is more suitable for the objective of our study. Third, en face imaging provides flattened coronal plane images after adjustment for the eyeball curvature using the RPE or Bruch membrane as a reference plane, which is easier to interpret and to compare with other imaging modalities with coronal planes, such as FA or ICGA. Volumetric data sets can provide multiple images at various tissue levels, which is advantageous for following vessels located across multiple depths.

The treatment effects of PDT in patients with CSC likely result from short-term choriocapillaris hypoperfusion and long-term choroidal vascular remodeling, leading to reductions in choroidal congestion, vascular hyperpermeability, and extravascular leakage [23]. Many PDT studies seeking to treat CSC have primarily focused on SCT, which was largely attributed to the thickness of Haller’s layer. The SCT cannot reflect the short-term treatment effect, which primarily depends on changes in the choriocapillaris, which accounts for a small fraction of the SCT. In the present study, SCT correlated with the vascular proportions of Haller’s layer but not with the choriocapillaris or Sattler’s layer (Fig 2). Few studies have evaluated changes in the choroidal vasculature based on 2-dimensional ICGA images or cross-sectional OCT images; nevertheless, studies have shown overlap between all choroidal vascular layers, although changes in individual layers could not be evaluated [24, 25].

We objectively evaluated the choroidal vasculature at 3 different layers: the choriocapillaris, Sattler’s layer, and Haller’s layer. To the best of our knowledge, there have been no reports of the quantitative evaluation of the vascular proportions at 3 different layers in CSC and their changes after PDT. In the present study, the vascular proportion in the choriocapillaris showed an early decrease after HF-PDT, coincident with the disappearance of subretinal fluid accumulation. The vascular proportions of Sattler’s and Haller’s layers also showed subtle decreases in the early period but showed significant decreases at later periods. This finding indicates that it takes longer for larger choroidal vasculature to show structural changes, but the mechanism for this effect has not been elucidated. Previous studies evaluating choroidal vasculature changes after PDT using ICGA and OCT cross-sectional images also showed that narrowing of the dilated and congested choroidal vasculature was observed in the early period, but significant changes were observed later [24, 26]. The effect of PDT that varies among choroidal layers—more in the shallower vessels with smaller diameters and less in the deeper vessels with larger diameters [27, 28]—may also result in earlier changes in the choriocapillaris and later changes in Sattler’s layer and Haller’s layers. The importance of this study lies in its demonstration of differences in the vascular changes among the choroidal layers in CSC eyes before and after PDT, which provides insight into the pathogenesis of CSC and the treatment mechanism of PDT.

Persistent choriocapillaris hypoperfusion is one of the complications related to PDT, which is believed to be reduced with low-fluence PDT [29, 30]. However, whether HF-PDT causes persistent deleterious effects in large-caliber choroidal vessels is not yet known. In the present study, the choriocapillaris, Sattler’s layer and Haller’s layer exhibited decreases in vascular proportions after HF-PDT but vascular proportions similar to the untreated fellow eye at 1 year after HF-PDT. These data suggest that HF-PDT did not cause persistent choriocapillaris hypoperfusion or excessive vascular remodeling, which can result in atrophic changes in the large-caliber choroidal vessels.

The current study had several limitations, including its small sample size and retrospective design. First, the SCT and the choroidal vascular proportions were not obtained at exactly the same time in each session; thus, diurnal variations in the choroid may have distorted the results. However, all examinations occurred between 2 PM and 4 PM to minimize the amplitude of the variation. Second, en face images of each layer collected at follow-up visits may not represent the same depth location because the depth level of each vascular layer changes with the decrease in the SCT after HF-PDT. To overcome this limitation, en face images representing the same depth location to the previously adopted baseline image were collected by 3 independent investigators. Disagreements were resolved by a second review. In the second review, collected images were pooled and re-selected by 3 independent investigators in a blinded fashion. This second review was repeated until there was disagreement. Third, we obtained images with large scan areas, which might limit their resolution. However, the limitations with transverse resolution of en face imaging can be overcome by adjustments in the digital systems with incorporating the axial dimension of the choriocapillaris, which enhance the accuracy of the evaluation of the choriocapillaris [29]. A further study with a higher resolution and a protocol with more scans would be required to support the findings of the present study. Finally, the distinction between the vascular layers was not clear-cut. However, choriocapillaris, Sattler’s layers, and Haller’s layers are easier to divide in en face imaging due to the fact that the orientation of the images follows the anatomic distribution of the vessels, which provides more precise segmentation.

In conclusion, using en face images of SS-OCT, we found that CSC eyes had increased choroidal vascular proportions compared with those of healthy patients in the choriocapillaris, Sattler's layer, and Haller's layer, which decreased after HF-PDT. Increased choriocapillaris vascular proportion compared with that of the unaffected fellow eyes and its rapid decrease with the absorption of subretinal fluid after HF-PDT imply the underlying pathogenesis of CSC, whereas delayed vascular proportion changes in Sattler's and Haller's layers reflect the long-term effects of HF-PDT.

## Supporting information

S1 Appendix Changes in whole, stromal, and luminal areas in the total choroid, inner choroid layer, and outer choroid layer in CSC eyes after half-fluence photodynamic therapy (HF-PDT). (A) In the total choroid, the luminal and stromal area, and the whole total choroid area did not show a significant decrease at 6 weeks after HF-PDT (total luminal area: *P* = 0.162; total stromal area: *P* = 0.315; whole total choroid area: *P* = 0.205). At 6 months, only the total luminal (*P* = 0.046) and whole total choroid area (*P* = 0.042) showed significant decreases, not the total stroma area (*P* = 0.062). At 12 months, the total luminal (*P* = 0.007) and total stromal area (*P* = 0.008), and the whole total choroid area (*P* = 0.006) showed significant decreases. (B) The inner choroid showed no significant change in the luminal (6 weeks: *P* = 0.586; 6 months: *P* = 0.776; 12 months: *P* = 0.448) and stromal area (6 weeks: *P* = 0.590; 6 months: *P* = 0.782; 12 months: *P* = 0.513), and the whole inner choroid area (6 weeks: *P* = 0.585; 6 months: *P* = 0.777; 12 months: *P* = 0.466) after HF-PDT. (C) In the outer choroid, the luminal and stromal area, and the whole outer choroid area did not show a significant decrease at 6 weeks after HF-PDT (outer luminal area: *P* = 0.100; outer stromal area: *P* = 0.212; whole outer choroid area: *P* = 0.129) but decreased significantly at 6 months (outer luminal area: *P* = 0.046; outer stromal area: *P* = 0.027; whole outer choroid area: *P* = 0.042) and 12 months (outer luminal area: *P* = 0.004, outer stromal area: *P* = 0.004, whole outer choroid area: *P* = 0.003).
(TIF)

S1 File Dataset of study patients. (XLSX)
