# Supplementary figures and images for: Choroidal vascularity changes in idiopathic central serous chorioretinopathy after half-fluence photodynamic therapy
Source: PLoS One. 2018 Aug 27;13(8):e0202930. doi: 10.1371/journal.pone.0202930 (PMC6110491; doi:10.1371/journal.pone.0202930)

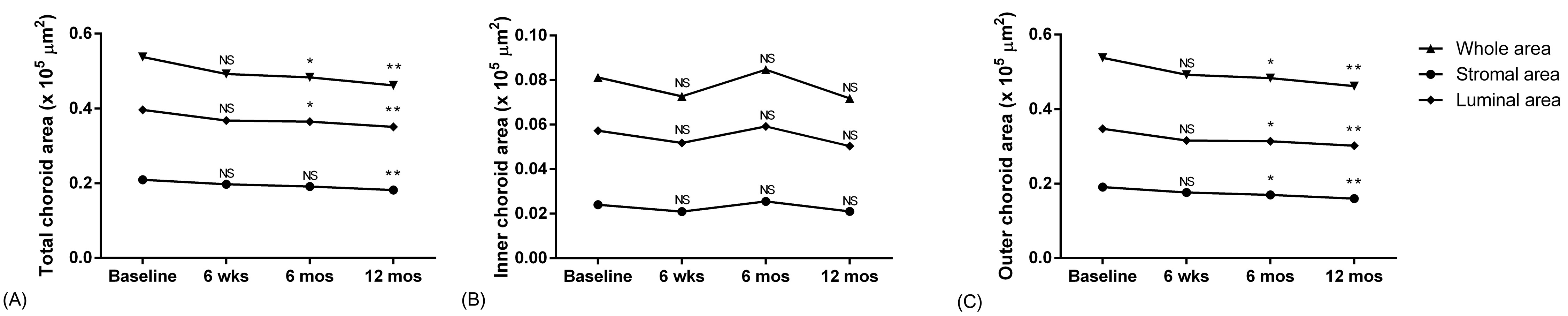

Supplement: S1 Appendix — (A) In the total choroid, the luminal and stromal area, and the whole total choroid area did not show a significant decrease at 6 weeks after HF-PDT (total luminal area: P = 0.162; total stromal area: P = 0.315; whole total choroid area: P = 0.205). At 6 months, only the total luminal (P = 0.046) and whole total choroid area (P = 0.042) showed significant decreases, not the total stroma area (P = 0.062). At 12 months, the total luminal (P = 0.007) and total stromal area (P = 0.008), and the whole total choroid area (P = 0.006) showed significant decreases. (B) The inner choroid showed no significant change in the luminal (6 weeks: P = 0.586; 6 months: P = 0.776; 12 months: P = 0.448) and stromal area (6 weeks: P = 0.590; 6 months: P = 0.782; 12 months: P = 0.513), and the whole inner choroid area (6 weeks: P = 0.585; 6 months: P = 0.777; 12 months: P = 0.466) after HF-PDT. (C) In the outer choroid, the luminal and stromal area, and the whole outer choroid area did not show a significant decrease at 6 weeks after HF-PDT (outer luminal area: P = 0.100; outer stromal area: P = 0.212; whole outer choroid area: P = 0.129) but decreased significantly at 6 months (outer luminal area: P = 0.046; outer stromal area: P = 0.027; whole outer choroid area: P = 0.042) and 12 months (outer luminal area: P = 0.004, outer stromal area: P = 0.004, whole outer choroid area: P = 0.003). (TIF) [file pone.0202930.s001.tif]
